# Hypoxia increases microbial carbon assimilation of taurine in a seasonally anoxic fjord

**DOI:** 10.1093/ismejo/wrag057

**Published:** 2026-03-17

**Authors:** Ömer K Coskun, William D Orsi, Ian P G Marshall, Katharina A Muschler, Nico Mitschke, Timothy G Ferdelman, Gonzalo V Gomez-Saez

**Affiliations:** Department of Earth and Environmental Sciences, Ludwig-Maximilians-Universität München, Richard-Wagner-Straße 10, Munich, 80333, Germany; Department of Earth and Environmental Sciences, Ludwig-Maximilians-Universität München, Richard-Wagner-Straße 10, Munich, 80333, Germany; GeoBio-Center^LMU^, Ludwig-Maximilians-Universität München, Richard-Wagner-Straße 10, Munich, 80333, Germany; Section for Microbiology, Department of Biology, Aarhus University, Ny Munkegade 114, Aarhus C, DK-8000, Denmark; Department of Earth and Environmental Sciences, Ludwig-Maximilians-Universität München, Richard-Wagner-Straße 10, Munich, 80333, Germany; Institute for Chemistry and Biology of the Marine Environment (ICBM), School for Mathematics and Science, Carl-von-Ossietzky Universität Oldenburg, Oldenburg, 26129, Germany; Department of Biogeochemistry, Max Planck Institute for Marine Microbiology, Celsius Straße 1, Bremen, 28359, Germany; Department of Earth and Environmental Sciences, Ludwig-Maximilians-Universität München, Richard-Wagner-Straße 10, Munich, 80333, Germany; GeoBio-Center^LMU^, Ludwig-Maximilians-Universität München, Richard-Wagner-Straße 10, Munich, 80333, Germany

**Keywords:** deoxygenation, dissolved organic sulfur, organosulfur, carbon cycle, qSIP, microbial ecology, taurine, methionine, fjord ecosystems

## Abstract

Hypoxic zones are expanding globally altering marine biogeochemical cycles. Within these low-oxygen regions, microbial communities play a key role in the production, degradation, and transformation of dissolved organic sulfur (DOS) compounds. Taurine is a bioavailable DOS compound widely utilized by marine microbes with a central role in nutrients exchange, energy production and biomass generation. However, in stratified water columns with varying oxygen conditions, the specific microbial taxa assimilating taurine as a carbon source remain poorly characterized. Here, we applied quantitative stable isotope probing (qSIP) experiments using ^13^C-labeled organosulfur compounds (taurine and methionine) and ^13^C-glucose to identify active microbial utilizers in oxic and hypoxic waters in the seasonally anoxic Mariager Fjord (Denmark, Kattegat Sea). Our qSIP results were supported by physicochemical measurements and geochemical data. Taurine-derived ^13^C-carbon was assimilated into microbial biomass exclusively under hypoxic conditions, primarily by *Flavobacteriaceae* (*Bacteroidota*), indicating that taurine serves as a carbon source only when oxygen is limited. ^13^C-taurine and ^13^C-methionine assimilation were strongly associated, suggesting a flexible metabolic strategy for utilizing organosulfur compounds in hypoxic waters. In oxic waters, ^13^C-methionine and ^13^C-glucose were assimilated by distinct taxonomic groups, dominated by *Bacteroidota* and *Verrucomicrobiota*, respectively. Overall, our study identifies active microbial communities assimilating organosulfur compounds under varying oxygen levels in the seasonally anoxic Mariager Fjord, providing new insights into key microbial processes in low-oxygen coastal systems.

## Introduction

Marine dissolved organic sulfur (DOS) represents the largest reservoir of organic sulfur in the ocean, exceeding particulate organic sulfur (POS) and sulfur associated with microbial and phytoplankton biomass [1, 2]. The majority of DOS in the ocean is produced by phytoplankton via the assimilatory sulfate (SO_4_^2−^) reduction (ASR) pathway [3]. During this process, SO_4_^2−^ is enzymatically reduced into a variety of organosulfur compounds via three principal routes: sulfolipid formation, cysteine synthesis, and sulfonation of organic compounds [3]. The resulting organosulfur metabolites, such as dimethylsulfoniopropionate (DMSP), dimethylsulfide (DMS), sulfur-containing vitamins (e.g. thiamin and biotin), and amino acids like methionine, cysteine, and taurine, play central roles in cellular metabolism, osmoprotection, redox regulation, and climate-relevant sulfur fluxes [3–7]. These DOS compounds are released into seawater through a combination of mechanisms, including active or passive export, viral lysis, protist grazing, and leakage during bloom senescence [3]. Many labile DOS compounds are remineralized within hours to days through the microbial loop [2], providing energy and biomass for heterotrophic microbial assemblages [4]. However, a significant portion escapes this recycling by being transported to greater depths via circulation or particle flux [8]. These exported compounds may resist biodegradation due to abiotic sulfurization [9, 10], and/or microbial metabolic limitations under low-oxygen conditions [11, 12]. Taurine, a sulfonate primarily derived from marine metazoans and phytoplankton [13], serves as an essential source of carbon, nitrogen, and sulfur for microbial energy production or biomass synthesis [4]. It contributes substantially to the heterotrophic carbon biomass production in the North Atlantic Ocean (16%–21%) [14] and supports microbial communities in anoxic sediments, where over 50% of bacteria are capable of taurine utilization [15]. Genes involved in taurine uptake and metabolism are widely distributed among marine prokaryotes [16–18]. Despite the ecological importance of taurine as a key organosulfur compound in the ocean, the identity and activity of its microbial degraders in low-oxygen environments remain poorly understood.

Since the middle of the 20^th^ century, dissolved oxygen (O_2_) levels in marine and coastal environments have declined due to anthropogenic activities leading to eutrophication and global warming [19], resulting in the rapid and widespread expansion of hypoxic (<63 μM O_2_; [20]) and anoxic zones in the water column [21]. These conditions are created from increased microbial respiration fueled by nutrient loading and reduced O_2_ solubility in warmer waters [22]. Although O_2_-depleted waters naturally occur in oceanic oxygen minimum zones (OMZs), anthropogenic activities have enhanced deoxygenation in coastal systems, including fjord ecosystems [23]. Fjords cover only ~0.1% of the global seafloor but they account for ~11% of the ocean’s organic carbon burial, largely due to their high sedimentation rates and steep geochemical gradients [24]. Many fjords are characterized by restricted water exchange between basin and coastal waters, leading to development of seasonally or even permanently hypoxic or anoxic bottom waters [25]. These hydrographic and geochemical conditions strongly influence microbial spatial organization, making fjords ideal systems for studying functional ecology in low-oxygen environments. Environmental gradients, such as varying oxygen concentrations, have been identified as primary drivers of prokaryotic community structure in fjord ecosystems [26–28]. For instance, distinct microbial communities and metabolisms were observed in a permanently anoxic fjord, with the ubiquitous SUP05 clade dominating the hypoxic layers [28]. As coastal deoxygenation intensifies due to climate change and increased eutrophication [21], understanding how microbial communities perform DOS and carbon cycling in fjord ecosystems becomes highly relevant.

**Figure 1:**
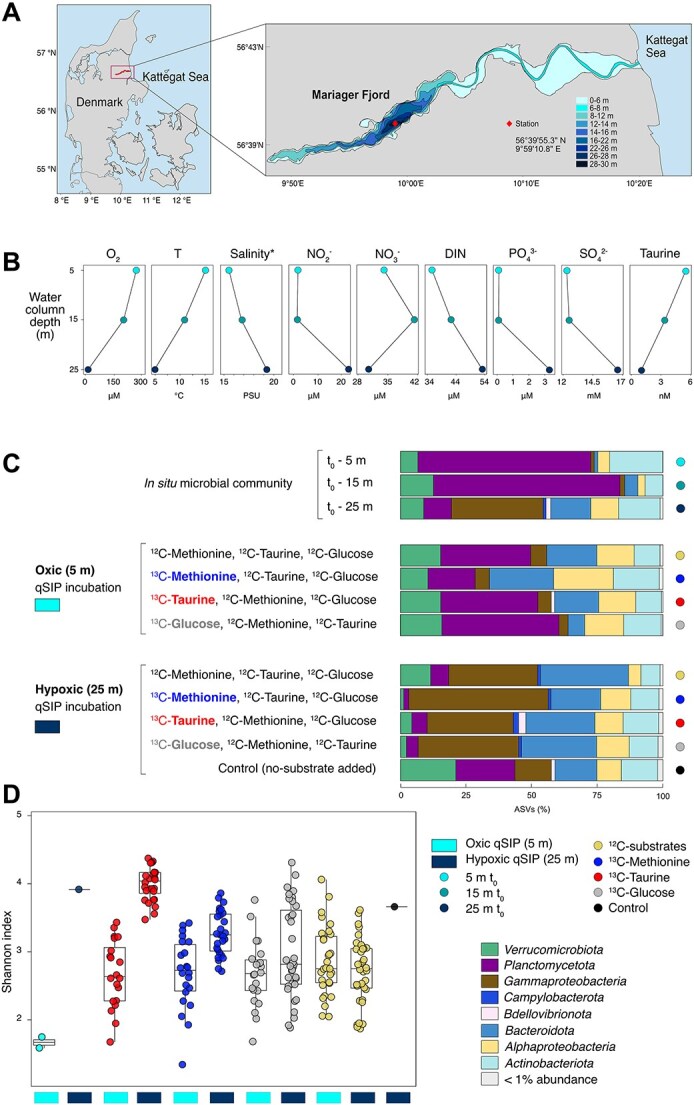
Study area, geochemistry and microbial community composition. (A) Map showing the location of Mariager Fjord and the sampling station (red). Fjord bathymetry was vectorized from: https://brakvand.com/smukkeste-fjord. (B) Geochemical profiles measured in water column samples collected at 5, 15 and 25 m depth. Dissolved inorganic nitrogen (DIN) contains total nitrate plus nitrite. (^*^): Salinity profile represents late May 2019 data obtained from a previously published article [29] (C) relative microbial community abundance based on 16S rRNA gene taxonomy at phylum/class level from untreated water column samples (t_0_), from oxic and hypoxic qSIP incubations with added ^13^C and ^12^C substrates, and control incubation with no-substrate addition. (D) Shannon diversity index of Mariager Fjord samples.

Mariager Fjord (Denmark, Kattegat Sea) is an example of a seasonally anoxic environment subject to deoxygenation [29–31]. Hypoxic and anoxic events are recurrent in Danish fjords, estuaries and open waters, primarily driven by seasonal water column stratification and algal growth [32, 33]. These events typically intensify during summer periods when warm weather conditions lead to a temporary stratification and increased respiration by marine organisms [34]. Mariager Fjord is connected to the Kattegat Sea by a shallow sill that limits deep-water exchange and facilitates the development of low-oxygen conditions [29] (Fig. 1A). As a result, Mariager Fjord serves as a valuable natural laboratory for investigating how biogeochemical processes respond to varying oxygen conditions. Although some research has focused on microbial community composition [31] and methane cycling [29], the microbial dynamics involved in taurine and organosulfur cycling in this system remain largely unexplored. In this study, we applied DNA-quantitative stable isotope probing (DNA-qSIP; [35]) in Mariager Fjord across a vertical oxic-to-hypoxic gradient in the water column. Our aim was to understand how changing dissolved oxygen concentrations influence microbial assimilation of ^13^C-taurine. Our results were compared to the assimilation of ^13^C-glucose and ^13^C-methionine. To target the key microbial assemblages involved in taurine cycling during the early development of hypoxia in spring [29], we sampled at oxic (5, 15 m) and hypoxic (25 m) depths, as oxygen concentration declined at the start of the summer season [29]. We followed a multisubstrate qSIP approach [35, 36] to assess microbial ^13^C-assimilation under defined incubation conditions. Namely, we incubated oxic and hypoxic samples (i) with all three substrates (taurine, methionine, glucose) at their natural isotopic composition (herein referred to as ^12^C-labeled); (ii) with an individual ^13^C-labeled substrate whereas the remaining substrates were provided at natural isotopic abundance; and (iii) without any added substrates [36]. Our qSIP results were supported by physicochemical measurements and geochemical data quantifying taurine, sulfate and nutrients concentration in the stratified water column of the fjord (Fig. 1).

## Materials and methods

### Water column sampling

Water column samples were obtained from the deepest part of the Mariager Fjord (Denmark, Kattegat Sea) (56°39′55.3″ N, 9°59′10.8″ E) on May 12th 2023 at depths of 5, 15, and 25 m using two 5 L Niskin Plastic Water Sampler PWS (Hydro-Bios, Germany) (Fig. 1A). Immediately after sampling, an aliquot of 100–200 mL was transferred to an acid-precleaned glass beaker for measurement of temperature and dissolved oxygen (O_2_) on board. We used temperature sensor probes (1.5 mm diameter, 100 mm length, TDIP15, Pyroscience, Germany) and oxygen optodes (3 mm diameter, 100 mm length, with optical isolation OXROB10, Pyroscience, Germany) connected to a previously calibrated pocket oxygen-meter (FireSting-GO2, FSGO2, Pyroscience, Germany).

Subsamples were then taken for analysis of nitrate (NO_3_^−^), nitrite (NO_2_^−^), nitrogen oxides (NO_x_), phosphate (PO_4_^3−^), SO_4_^2−^, hydrogen sulfide (H_2_S), and taurine. Total NO_3_^−^ and NO_2_^−^, NO_2_^−^, and PO_4_^3−^ were measured with a QuAAtro39 autoanalyzer (Seal Analytical, Germany) after the method based on [37]. For H_2_S, SO_4_^2−^, and taurine, 30 mL of water samples were syringe filtered (0.22 μm GHP filters Pall Life Science, NY, USA). H_2_S and SO_4_^2−^ samples were fixed with 5% zinc chloride. Taurine samples were immediately frozen. SO_4_^2−^ was determined by suppressed ion chromatography using a Metrohm 761 Compact IC (Metrohm AG, Switzerland; Metrohm A Supp 5 column; 3.2 mM Na_2_CO_3_ and 1 mM NaHCO_3_ eluent; 20 μL sample loop). Total dissolved H_2_S, was measured using the methylene blue method [38]. Taurine was measured using a Waters Aquity H Class UPLC with fluorescence detection by adapting the ortho-phthalaldehyde (OPA) pre-column derivatization method HPLC [14, 39–41]. More information on taurine measurements is included in the Supplementary Material.

The initial microbial composition was characterized from duplicate samples of 5 L of fjord water from each depth, which were immediately filtered through a 0.22 μm hydrophilic polyethersulfone (PES) filters (Millipore Express, Merck, Germany) using a peristaltic field pump (Masterflex E/S 07571–05, Cole Parmer, IL, USA) after the recovery of the Niskin bottles on deck, within 10 min after sampling. After filtration, samples were stored on ice during transport to the Aarhus University (Denmark) and subsequently kept at −20°C until DNA extraction.

### Experimental setup for ^13^C incubations

For qSIP incubations, 1 L autoclaved borosilicate glass bottles (VWR, Germany) were filled to the 1 L mark with the water column samples collected from 5 and 25 m depth, and the bottle caps were closed tightly until the addition of ^13^C and ^12^C-labeled substrates. The bottles had ~160 mL headspace filled with atmospheric air. Within two hours of collection, each bottle received a mixture of 40 nM methionine, glucose, and taurine, where only one substrate was ^13^C-labeled and the other two were added in their unlabeled (^12^C) form. The bottles were incubated statically (without agitation) in the dark for 48 h at temperatures close to original conditions (15°C and 4°C for 5 and 25 m depth, respectively; Fig. 1B). Incubation time was selected to allow sufficient ^13^C incorporation into DNA while minimizing cross-feeding. Comparable incubation durations have been successfully applied for SIP analyses of dissolved organic substrates (e.g. [42, 43]), highlighting that short-period incubations are suitable for identifying active carbon-assimilating taxa in marine water columns. The multisubstrate qSIP approach was used to systematically investigate the microbial carbon assimilation dynamics while maintaining identical experimental conditions across samples [36]. As a common practice in qSIP incubations, a single bottle only received 40 nM of ^12^C-labeled experiments (methionine, taurine, and glucose) to set a reference point for comparison with ^13^C-labeled experiments [35]. We also kept incubations without substrate addition from the hypoxic water (Fig. 1C). Throughout the manuscript, ^13^C-incubations are referred to as ^13^C-glucose, ^13^C-methionine and ^13^C-taurine treatments, corresponding to the specific substrate that was labeled in each case. To ensure comparability in the incorporation rates among the added compounds in our qSIP incubations, we used identical substrate concentrations. The natural concentrations of the added substrates differ across the ocean. Methionine concentration ranges between 0.2 and 0.69 nM [44], whereas taurine can reach up to 30 nM in coastal settings [45]. The free glucose concentrations vary between 1.5 and 55 nM in the upper 200 m of the water column in North Pacific [46]. We acknowledge that the addition of 40 nM of each substrate in our incubations is within the natural glucose range, slightly above the taurine concentrations and significantly higher than natural methionine levels. However, we were unable to set our incubations to the lowest natural concentration among our used substrates (methionine) as the added concentrations were already approximately three orders of magnitude lower than the recommended substrate concentrations of ca. 50 μmol ^13^C per g sediment [47], as suggested for sufficient DNA-SIP labeling. Incubated seawater samples were filtered using the same PES filters and peristaltic field pump than for the initial microbial community composition (see above). Filters were immediately stored at −20°C and transported at that temperature to the laboratory in Ludwig-Maximilians-Universität (LMU) Munich (Germany) where we performed the rest of laboratory work.

### DNA extraction

DNA was extracted from both untreated samples (t_0_) and qSIP experiments as described previously [48]. Briefly, each 15 mL Falcon tube containing the filters was supplemented with lysing matrices from three 2 mL Lysing Matrix E tubes (Mp Biomedicals, OH, USA). A 4 mL volume of sterile-filtered sucrose ethylenediaminetetraacetic (EDTA) lysis buffer (containing 0.75 M sucrose, 0.05 M Tris-Base, 0.02 M EDTA, 0.4 M NaCl, adjusted to pH 9 with NaOH) and 100 μL 10% w/v sodium dodecyl sulfate (SDS) were then added. The samples were subjected to bead beating for 40 sec at 4 m/s using a Fast-Prep 5G homogenizer (MP Biomedicals, OH, USA), followed by heating at 99°C for 2 min. After heating, 25 μL of 20 mg/mL proteinase K was added, and the tubes were incubated overnight at 55°C. Buffer solution containing DNA were then centrifugated at 4700 RPM and the supernatant was transferred to Amicon filters (30 KDa; Millipore, Merck, Germany). DNA was extracted and purified from concentrate using DNeasy Blood and Tissue Kit (Qiagen, Germany). The extracted DNA was suspended in 200 μL of deionized, ultrapure DEPC-treated, nuclease-free water, and its concentration was measured fluorometrically using a Qubit 3.0 fluorometer (Invitrogen, Eugene, OR, USA).

### Density gradient centrifugation, gradient fraction and qPCR

Density gradient centrifugation was applied according to established SIP protocols [35, 48]. For each SIP experiment, three independent ultracentrifugation runs were conducted as technical SIP replicates using 50 μL aliquots of the same DNA extract. This design allowed us to quantify methodological variability in density gradient ultracentrifugation and fractionation, which represents a major source of methodological uncertainty in qSIP workflows [15, 48]. Due to logistical problems, biological replication was not feasible as including them would triple the required amount of 1 L bottles. Hence, replication focused on capturing technical reproducibility of isotope incorporation measurements. The resulting variability was incorporated into the qSIP calculations as 90% confidence intervals following established approaches [35]. Fraction density intervals were chosen between 0.005 and 0.007 g mL^−1^, consistent with the empirically determined detection threshold for reliable isotope enrichment (>90% sensitivity and >95% specificity [49]). Universal primers (515F-Y/806RB [50, 51]) targeting the V4 hypervariable region of 16S ribosomal RNA (rRNA) were used in all qPCRs to evaluate microbial abundances in the extracted DNA samples. All qPCRs were performed in a CFX Connect real-time PCR (Bio Rad, Hercules, CA, USA) as described previously [15, 48, 52].

### DNA sequencing and qSIP analysis

To determine if there was an ^13^C-enrichment in the microbial DNA, the qPCR-derived starting quantity (SQ) values, representing the number of 16S rRNA gene copies in each SIP fraction, were plotted against their corresponding buoyant densities for both experimental and control incubations (Fig. S1). To capture the full range of isotopic enrichment, 7–14 fractions from each incubation were selected for DNA-sequencing, spanning the peak and adjacent flanking regions (Fig. S1, Data S2). Selected samples were uniquely tagged in qPCR. Each qPCR assay was performed in two technical replicates by doubling the reaction volume and distributing it into two wells containing identical reagents and template DNA to minimize the pipetting and amplification errors. The barcoded amplicons were purified using Qiagen gel extraction kit (Qiagen, Hilden, Germany) and diluted to 1 nM prior to DNA sequencing on a MiniSeq System at LMU Munich (Germany) following an established protocol [53]. One set of incubations (96 samples, DNA pool at 3 nM) was sent for DNA sequencing using a MiniSeq System (Illumina, San Diego, CA, USA) at Procomcure Biotech company (Thalgau, Austria). Samples obtained from western Gotland Basin (58°10′17.89″N; 18°14′09.06″E) from the expedition onboard the RV Skagerak [54] were sequenced together with the samples from Mariager Fjord (see Supplementary Material).

Quality filtering, read assembly, and contaminant removal from 16S rRNA gene amplicon sequences were carried out using established protocols described in [55]. In brief, amplicon sequence variants (ASVs) were generated using QIIME2 [56]. The initial dataset contained 6717 ASVs which were further quality filtered as follows (Table S1). First, low-abundant 4447 ASVs ( <10 reads across whole fractions) were filtered from the dataset, including common contaminants (500 ASVs). Contaminants were flagged based on two sources: (i) known reagent and lab contaminants determined by [57] and (ii) previously sequenced contaminants from our lab facilities at LMU Munich (Germany) [53], which were deposited to NCBI with accession numbers (SRR13102744-SRR13102755) [55]. Second, common contaminants still retained in the dataset (170 ASVs) such as *Ralstonia*, *Variovorax*, or *Streptococcus* [57] were deleted. Reads belonging to eukaryotic organelles, including mitochondria and chloroplasts, were also removed in this step. These sequence reads comprised 2.9% of the whole dataset. Third, only ASVs represented with >10 reads in total in each replicate within the same experimental setup were selected for further analysis because low abundance taxa might create artificial variations in qSIP calculations [58]. Based on these selection criteria, the final datasets included 42, 44 and 43 ASVs for ^13^C-taurine, ^13^C-methionine, and ^13^C-glucose incubations at oxic conditions, respectively, whereas 87, 86 and 84 ASVs were present in the same incubations at hypoxic conditions (Table S1). The ^13^C-excess atom fraction (EAF) values for each ASVs were calculated following the protocol previously described [35] using the HTSSIP R package [59]. An ASV was considered a ^13^C incorporator if its 90% lower confidence interval was greater than zero [35] (Data S1). Statistical analysis, including non-metric multidimensional scaling (NMDS) and graphic plotting were performed using the phyloseq [60] and vegan [61] packages in R Studio. To check whether there is a linear relationship in our data, we used raintest() function in lmtest package [62] and confirmed that our data met the assumptions of linearity. Linear regressions were performed using the lm() function in R Studio to evaluate relationships between isotope incorporation patterns. Model performance was reported as *R^2^*, *F*, and *P* values. Further details on molecular analyses and bioinformatic workflow (such as PCoA analyses) are provided in the Supplementary Material.

## Results

### Geochemistry

The water column of Mariager Fjord (Denmark, Kattegat Sea; Fig. 1A) exhibited clear stratification. Temperature profiles showed a thermocline, with temperatures dropping from 15°C at the surface 5 m waters to 11°C at 15 m depth, and further down to 5°C at 25 m water depth (Fig. 1B). Salinity inversely mirrored temperatures, with surface salinities of 16 increasing to 19 at 25 m water depth (Fig. 1B). Dissolved O_2_ levels decreased from close to saturation concentrations of 272 μM O_2_ (95% saturation) at the surface (5 m) down to 14 μM O_2_ (4% saturation) in the bottom waters at 25 m (Fig. 1B). The deeper O_2_ concentrations indicate the presence of hypoxic conditions (<63 μM O_2_; [20]) at 25 m. Throughout this study, incubations from 5 m depth are referred to as “oxic” and those from 25 m depth as “hypoxic,” corresponding to the dissolved O_2_ levels measured in Niskin subsamples immediately after sample recovery. For this reason, SIP incubations were performed at temperatures close to natural conditions (15°C and 4°C for 5 and 25 m depth, respectively). In the oxic surface layer, NO_3_^−^ concentration was 34 μM and rose to 41 μM at 15 m, before decreasing to 30 μM under hypoxic conditions. NO_2_^−^ remained relatively low and constant at 1.4 and 1.2 μM at 5 and 15 m, respectively, but with a concentration of 23 μM at 25 m water depth, NO_2_^−^ composed close to half of the total fixed N pool. Conversely, NO_3_^−^ (30 μM) was depleted at 25 m depth. PO_4_^3−^ concentrations were low and depleted relative to expected Redfield N:P ratios in the surface and 15 m samples, whereas dissolved P at depth (3.0 μM) was closer to Redfield (16:1, N:P). SO_4_^2−^ concentrations mirrored salinity, going from 12 mM at the surface to 17 mM at depth. H_2_S remained below the detection limit (1 μM) in all samples. Taurine exhibited a steady decline in concentration with depth, with concentrations of 5.5 nM at 5 m, 3.4 nM at 15 m falling below detection at 25 m water depth (1.0 nM; Fig. 1B).

### Microbial community composition

The microbial community composition (16S rRNA gene) of water column in the surface oxic waters was dominated by amplicon sequence variants (ASVs) affiliated with *Planctomycetota* (66% of total sequences at 5 m depth), followed by *Actinobacteriota* (20%) and *Verrucomicrobiota* (7%) (Fig. 1C). At 15 m depth, the microbial communities were similar. *Planctomycetota* remained dominant (71%), with an increase of *Verrucomicrobiota* (13%) and a decrease of *Actinobacteriota* (7%) compared to oxic water column. In contrast, in the hypoxic bottom conditions, there was a clear change in microbial community composition (Fig. 1C). *Gammaproteobacteria* (34%) became the dominant group, followed by *Actinobacteriota* (16%) and *Bacteroidota* (15%). *Planctomycetota* (11%) in the hypoxic waters drastically decreased compared to its dominance at oxic conditions and at 15 m depths. PCoA analysis also reflected the significant differences in the microbial compositions between the oxic and hypoxic water columns (Fig. S2). Shannon diversity index indicated that microbial diversity was significantly higher under hypoxic than under oxic conditions, except in fractions received ^12^C-substrates (Fig. 1D; Fig. S2). Although compositional variation in microbial communities was observed in oxic and hypoxic incubations relative to those of original field samples, the differences were not significant (Fig. 1C; permutational multivariate analyses of variance (PERMANOVA), oxic vs oxic-initial: *R*^2^ = 0.65, *F* = 5.64, *P* = 0.20, hypoxic vs hypoxic initial: *R*^2^ = 0.63, *F* = 5.04, *P =* 0.20). In contrast, a substantial change was observed in post-incubation communities between oxic and hypoxic conditions (Fig. 1C; PERMANOVA, *R*^2^ = 0.71, *F* = 14.6, *P =* 0.02). Additionally, the control bottle (hypoxic incubation) clustered closely to the other hypoxic samples (Fig. S2), there were only weak compositional differences between the no-substrate-added control bottle and substrate-amended hypoxic incubations (Fig. 1C). The ^13^C-glucose incubation showed no significant separation from the control (*R*^2^ = 0.04, *F* = 1.3, *P =* 0.13), whereas ^13^C-methionine and ^13^C-taurine incubations showed small but statistically detectable differences (*R*^2^ = 0.12, *F* = 3.57, *P =* 0.04 and *R*^2^ = 0.06, *F* = 1.76, *P =* 0.03, respectively). After incubations, *Planctomycetota* still dominated the microbial community (albeit a significant decrease in all incubations; 33%) under oxic conditions, whereas *Bacteroidota* (17%), *Alphaproteobacteria* (17%) and *Verrucomicrobiota* (14%) substantially increased (Fig. 1C). In contrast, in hypoxic waters, *Gammaproteobacteria* (39%) and *Bacteroidota* (27%) increased compared to untreated samples, whereas *Actinobacteriota* (11%) and *Planctomycetota* (5%) decreased (Fig. 1C). The incubation without added substrate from hypoxic waters contained mainly *Planctomycetota* (23%) and *Verrucomicrobiota* (21%), followed by *Bacteroidota* (16%) and *Actinobacteriota* (14%) (Fig. 1C).

### ^13^C-labeling in qSIP incubations

In our qSIP experiments at both depths, the incorporation of the ^13^C-labeled substrates into microbial DNA was determined by slight density shifts in the buoyant DNA density (Fig. S1). In the oxic water, DNA of incubations with ^13^C-methionine and ^13^C-glucose had slightly higher weighted average densities (1.6832 ± 0.0007 and 1.6859 ± 0.005 g/mL, respectively) compared to control experiment (1.6813 ± 0.0022 g/mL), whereas incubations with ^13^C-taurine exhibited average density at 1.6812 ± 0.005 g/mL similar to control incubation (Fig. S1). In contrast, at hypoxic conditions, ^13^C-taurine and ^13^C-glucose incubations resulted in higher weighted average densities at 1.684 ± 0.0024 and 1.6818 ± 0.002 g/mL compared to control experiment (1.680 ± 0.0007 g/mL). In contrast, incubation amended with ^13^C-methionine showed a density at 1.6797 ± 0.0014 g/mL, overlapping with average density in control experiments (Fig. S1).

### ^13^C-labeling of ASVs

**Figure 2:**
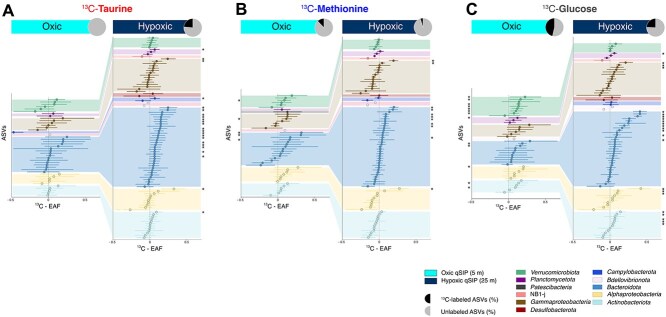
^13^C-excess atom fraction (^13^C-EAF) in the 16S rRNA genes of ASVs after 48 h of incubation with (A) ^13^C-taurine, (B) ^13^C-methionine and (C) ^13^C-glucose. Each panel shows ^13^C-EAF values for ASVs from oxic (left) and hypoxic (right) conditions. Points represent the median EAF values for each ASV across the three replicates, with error bars corresponding to 90% confidence intervals across the replicates. The ASVs are arranged vertically, from the highest to the lowest EAF values. The x-axis shows the percentage of ^13^C-labeled carbon atoms incorporated into the 16S rRNA genes for each population. ASVs are colored based on their phylum (or class, *Proteobacteria*) for taxonomic identification. The pie charts correspond to the relative abundance of ^13^C-labeled ASVs (black) within each treatment. EAF values that are positive and have confidence intervals that do not overlap zero (dashed lines) are considered statistically significant. The significantly ^13^C-labeled ASVs are represented with an asterisk (^*^).

The extent of ^13^C-labeling in ASVs was determined by analyzing the excess ^13^C-atom fraction (^13^C-EAF) in the v4 hypervariable region of 16S rRNA gene of each detectable ASV within the samples [35, 48] (Fig. 2, Data S1). The number of ASVs detected in qSIP incubations from hypoxic conditions was twice as high as those from oxic conditions (Fig. 2). ^13^C-labeled microbial communities exhibited varying degrees of substrate utilization in our qSIP incubations. Under oxic conditions, a total of 13 and 5 ASVs were identified assimilating ^13^C-glucose and ^13^C-methionine, respectively (Fig. 2). The relative abundance of ^13^C-labeled ASVs in the ^13^C-glucose incubations in the respective dataset was nearly four times higher than in the ^13^C-methionine incubations. However, in oxic waters, none of the microbial communities showed active assimilation of ^13^C-taurine as a carbon source. Conversely, under hypoxic conditions, all three substrates tested in this study were assimilated with different distinct patterns of microbial incorporation. In the incubations from the hypoxic layer amended with ^13^C-taurine and ^13^C-glucose, the same number of 23 ASVs exhibited significant ^13^C-assimilation, accounting for 23 and 24% of the total community relative abundance, respectively (Fig. 2, Data S1). In contrast, ^13^C-methionine was incorporated by a smaller number of ASVs (11 in total) compared to the other incubations, which accounted for 5% of the total microbial communities. These findings showed that despite relatively lower O_2_ concentrations in the hypoxic layer, the number of microbial taxa assimilating the added substrates was increased relative to oxic conditions, whereas the relative abundance of ^13^C-labeled taxa decreased in ^13^C-methionine and ^13^C-glucose incubations (Fig. 2, Data S1). In contrast, ^13^C-taurine was the only substrate with microbial assimilation exclusively in hypoxic waters (Fig. 2).

**Figure 3:**
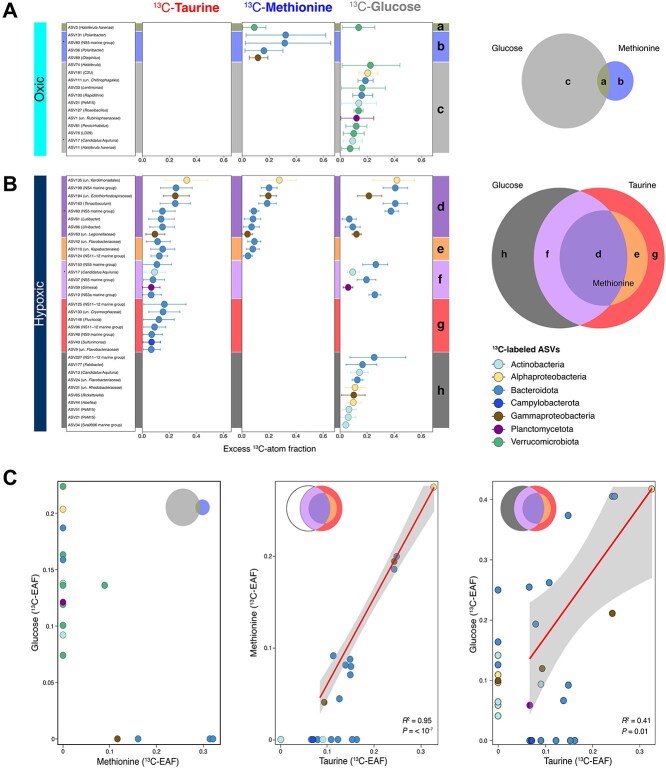
^13^C-EAF of significantly ^13^C-labeled taxa across oxic (A) and hypoxic (B) incubations, and comparison between different ^13^C-substrate treatments (C). (A-B) ^13^C-EAF of carbon-assimilating ASVs in the oxic (A) and hypoxic (B) incubations are shown with taxonomic information in parenthesis. These ^13^C-labeled ASVs are grouped based on their occurrences between the incubations (see Venn diagrams on the right side). Each point representing ^13^C-EAF of ASVs are colored according to their phylum/class level taxonomy. (C) Left: the ^13^C-EAF pattern of ASVs in the oxic incubation. There was no ^13^C-labeling observed in the ^13^C-taurine incubations from oxic conditions. Center: ^13^C-EAF relationship among ^13^C-labeled taxa in ^13^C-taurine and ^13^C-methionine incubations from the hypoxic conditions. Right: The ^13^C-EAF pattern of ASVs between ^13^C-taurine and ^13^C-glucose incubations in the hypoxic conditions. The linear regression line (red) with corresponding adjusted *R*^2^ and 95% confidence interval (gray area) is shown for panel C, where multiple shared ^13^C-ASVs are present.

We further provided an ASV-level comparison between the datasets with distinct groupings (Fig. 3). In oxic conditions, a single ASV (ASV3) affiliated with *Haloferula* (*Verrucomicrobiota*) utilized both ^13^C-methionine and ^13^C-glucose (“a” = 1, Fig. 3A). Exclusive ^13^C-methionine utilization at oxic waters was carried out by ASVs belonging to *Bacteroidota* and *Gammaproteobacteria* (“b” = 4, Fig. 3A), whereas ^13^C-glucose incubations resulted in a higher number of unique ^13^C-labeled ASVs (“c” = 12, Fig. 3A), in which half of them were associated with *Verrucomicrobiota*. In hypoxic waters, a total of eight ASVs exhibited significant incorporation with all the ^13^C-labeled substrates (“d” = 8, Fig. 3B). These included five distinct ASVs belonging to *Bacteroidota* genera, NS4 and NS5 marine group, *Tenacibaculum*, *Lutibacter*, and *Ulvibacter*. In all hypoxic incubations, the highest ^13^C-labeling was detected in ASV135 affiliated with unassigned genus of *Kordiimonadales* (between 0.27–0.41, median ^13^C-EAF; *Alphaproteobacteria*). In addition, three ASVs belonging to *Bacteroidota* genera (NS11–12 marine groups and unassigned *Flavobacteriaceae* and unassigned *Kapabacteriales*) assimilated ^13^C-labeled DOS substrates but no ^13^C-glucose (“e” = 3, Fig. 3B). ASVs utilizing both ^13^C-taurine and ^13^C-glucose were related with NS5 and NS3a marine group (*Bacteroidota*), *Candidatus* Aquiluna (*Actinobacteriota*), and *Gimesia* (*Planctomycetota*) (“f” = 5, Fig. 3B). Although no ASVs exclusively exhibited ^13^C-methionine assimilation in hypoxic waters, there were many unique ASVs utilizing either taurine or glucose (“g” = 7, taurine exclusive; “h” = 10, glucose exclusive). ASVs affiliated with *Bacteroidota* dominated the ^13^C-labeling in ^13^C-methionine, ^13^C-taurine, and ^13^C-glucose incubations, accounting for more than half of the active utilizers (Fig. 3). The utilization of at least two substrates in qSIP incubations under different oxygen conditions enabled a comparison of ASVs based on their ^13^C-EAF pattern (Fig. 3C). Although distinct ASVs were responsible for ^13^C-glucose and ^13^C-methionine assimilation in oxic conditions, there were many shared ASVs in the incubations performed under hypoxic conditions. A strong linear relationship was observed between ^13^C-methionine and ^13^C-taurine utilization in those showing assimilation of both substrates (linear regression; Adjusted-R^2^ = 0.95, *P =* 10^−7^), suggesting that ASVs efficiently metabolized both DOS compounds (Fig. 3C). This relationship further showed that ^13^C-taurine was more preferentially utilized over ^13^C-methionine as a carbon source. Moreover, ^13^C-taurine- and ^13^C-glucose-assimilating ASVs were weakly associated (linear regression; Adjusted-*R*^2^ = 0.41, *P =* 0.01) (Fig. 3C).

## Discussion

### Microbial taurine assimilation is limited to hypoxic conditions

Differences in ^13^C-substrate assimilation between oxic and hypoxic qSIP incubations were characterized by a greater number of carbon-assimilating taxa in hypoxic waters relative to oxic waters. Specifically, taurine-derived ^13^C-carbon uptake was detected only in hypoxic samples in our qSIP samples (Fig. 2A, 3), suggesting that taurine can serve as an important carbon source for microaerophilic microbial communities in low-oxygen coastal systems. This finding was further supported by geochemical measurements of taurine, with an observed depletion of taurine concentration under hypoxic conditions compared to oxic layers (Fig. 1B). Taurine metabolism has been documented across a wide range of oxygen regimes in environmental studies, including oxic marine waters [14], OMZs [63], and anoxic marine sediments [15], indicating its wide ecological importance. Culture-based studies similarly demonstrated a wide occurrence of aerobic taurine utilization [64]. Furthermore, anaerobic taurine utilization has been observed in *Alcaligenes* sp. strain NKNTAU (*Gammaproteobacteria*), where taurine-derived carbon was assimilated into biomass via NO_3_^−^ reduction and SO_4_^2−^ production [65]. Our qSIP approach showed that taurine assimilation in Mariager Fjord occurred exclusively under hypoxic conditions, suggesting that microbial taurine utilization may be more active under low-oxygen waters.

Many aerobic microorganisms are known to utilize taurine as a carbon and energy source in uncultured [14] and cultured studies [64], both in oxygenated marine and laboratory settings. However, the absence of detectable ^13^C-taurine assimilation in our qSIP data from oxic conditions (Fig. 2A) may reflect microbial adaptation to fjord-specific conditions rather than a general metabolic limitation. Unlike open ocean settings dominated by SAR11, *Cyanobacteria* and other aerobic heterotrophs actively metabolizing taurine [14, 66], Mariager Fjord displays a distinct microbial community shaped by restricted water exchange, periodic stratification, salinity gradients, and different water sources entering the system [29, 31] (Fig. S2A). These physical and hydrographic conditions may favor microbial assemblages adapted to fluctuating O_2_, leading to lower community diversity in the oxic layer and enrichment of *Bacteroidota* and *Gammaproteobacteria* in hypoxic waters, where diversity was moderately higher (Fig. 1C; Fig. S2B). In comparison, other fjord settings with limited water exchange such as Saanich Inlet and Roskilde Fjord [28, 67], exhibited higher microbial diversity (Fig. S2B) and distinct communities (Fig. S2A). In fact, microbial diversity from the hypoxic water column in Saanich Inlet showed slightly higher diversity in anoxic than oxic waters, a pattern consistent with our observations in Mariager Fjord (Fig. S2). PCoA analysis revealed that Mariager Fjord water samples were clustered separately from those of open-ocean and large fjord settings, likely exhibiting its unique hydrographic characteristics (Fig. S2A). Moreover, microbial communities from hypoxic waters of Mariager Fjord were clearly different than those in the oxic layers, including oxic surface waters in Roskilde Fjord (Denmark) (PERMANOVA; *R*^2^ = 0.42–0.58, *P =* 0.001; Fig. S2A), although, these samples appeared closely positioned in the PCoA ordination. Despite the different oxygen conditions between these fjords, salinity levels were nearly identical (ca. 19; Fig. 1B, Data S3), suggesting that salinity may play a stronger role than oxygen concentration in shaping the microbial community composition. Indeed, seasonality and salinity levels have been shown to be key environmental factors, controlling the microbial community structure in the Baltic Sea, Kattegat, and Skagerrak [68]. Altogether, our observed ^13^C-taurine assimilation under hypoxia likely suggests the ecological adaptation of Mariager Fjord communities to seasonal deoxygenation.

Phylogenetically dispersed groups including *Deltaproteobacteria*, *Gammaproteobacteria*, SAR11, and *Thaumarchaeota* have the genomic potential for taurine uptake across a range of environments [14, 15, 64, 69, 70]. In this study, we directly linked community-wide microbial diversity and ^13^C-taurine assimilation in the fjord water column (Fig. 1 and 2). By using ^13^C-labeled taurine to determine taxon-specific active microbial communities, we found that 75% of the ^13^C-labeled ASVs (17 out of 23 ASVs; Fig. 2 and 3A) assimilating ^13^C-taurine were affiliated with groups belonging to the phylum *Bacteroidota* (Fig. 2A and 3B). *Bacteroidota* is a frequently abundant bacterial phylum in OMZs [71] and can dominate hypoxic sediments [72]. Several members of this phylum can grow under facultative anaerobic or microaerophilic conditions, particularly in estuarine sediments subject to fluctuating oxygen [73]. This suggests a high degree of adaptability to changing redox conditions [72]. Furthermore, *Bacteroidota* are capable of degrading complex high-molecular-weight organic matter in marine environments, suggesting high metabolic versatility [74]. *Bacteroidota* strains are commonly associated with surface algal blooms, especially degrading algal-derived polysaccharides [75, 76]. Thus, our findings showed that *Bacteroidota* can be important assimilators of taurine and may contribute to taurine and organosulfur cycling in other ecologically similar fjord and low-oxygen coastal systems.

### Effects and potential limitations of our SIP incubations

Our results, and other similar SIP experiments, should be interpreted as potential/relative substrate utilization under bottle-incubation conditions rather than quantitative *in situ* rates or definitive depth-resolved oxygen effects. In our setup, the headspace in the bottles during the incubation likely resulted in a vertical oxygen gradient. By the time of sampling, in May 2023 (Fig. 1), the *in situ* conditions of Mariager Fjord presented hypoxic bottom waters with low oxygen concentration, connected to seasonal deoxygenation happening in the fjord [29] (Fig. 1). High nitrite concentrations in hypoxic waters (Fig. 1B) suggested that incomplete nitrification (ammonium to nitrite) happened shortly before sampling. Based on these geochemical considerations, it is highly likely that the microbial communities in the deep waters had been experiencing low but consistent exposure to dissolved oxygen, a characteristic of fjords that experience hypoxic conditions. Based on diffusion modeling [77] and microbial respiration rates from nearby fjords (30–400 nmol O_2_ L^−1^ h^−1^; [78]), we estimated that 80–90% of the water volume in the hypoxic water bottles likely remained hypoxic to near-anoxic over 48 h of incubation (Fig. S3). We did not measure oxygen during the SIP incubations and therefore, we cannot know for sure what the exact oxygen concentration inside each bottle was. Even applying high respiration fjord rates from the literature (400 nmol O_2_ L^−1^ h^−1^; [78]), the deeper water in the flask could have become anoxic after ca. 35 h. Thus, we suggest that a large portion of our incubated volume remained hypoxic for most of our 48 h period. This was consistent with the qSIP results, where we observed a clear contrast in carbon assimilation patterns of microbial communities between the oxic and hypoxic qSIP incubations (Fig. 2). If the hypoxic flasks had become fully oxygenated, we would expect microbial patterns to look similar to oxic incubations. Instead, the hypoxic incubations showed clear differences in ^13^C-carbon assimilation by taxa adapted to low-oxygen conditions (Fig. 2, Data S1). Specifically, many of the ^13^C-labeled ASVs in the hypoxic incubations were more abundant in the original microbial composition in hypoxic waters than in the oxic layer (Fig. 2), suggesting that they remained metabolically active under incubation conditions independently of oxygen contamination. Furthermore, comparison of the communities in the hypoxic qSIP incubations to the no-substrate control and the initial microbial community composition (Fig. 1C, Fig. S2) showed that “bottle effects” and addition of substrates did not greatly impact the community composition in the SIP incubations during our experiment.

Another limitation to every SIP incubation is linked to the concentration of the substrate. In our study, we added 40 nM concentration of each substrate to compare the assimilation at the same concentration. This concentration is higher than natural values for taurine and methionine but similar to glucose (see Methods). Concentrations mirroring natural concentrations, especially those of methionine, would have resulted in limitations of detectability. Nevertheless, the addition of 40 nM substrates does not appear to have greatly influenced the community composition as we did not observe in the SIP incubation great differences compared to the no-substrate control (Fig. 1C, Fig. S2). Furthermore, even though our use of technical replicates provides an assessment of the SIP method variability, we acknowledge that the lack of biological replication limits ecological extrapolations. Our multisubstrate qSIP approach was designed to make incubations comparable to each other. In other words, we expected that in every experimental bottle the microbes had equal access to all three substrates tested with only one ^13^C-labeled. Still, we cannot exclude interaction effects (e.g. preferential uptake, repression/priming) that might differ from single-substrate exposures. Altogether, we conclude that our qSIP data showing distinct ^13^C-labeling patterns across the different substrates, with different microbial groups incorporating different compounds (Fig. 2 and 3), suggested that ^13^C-substrate assimilation was selective and taxon-specific.

Another general limitation of SIP incubations is linked to the different conditions in a glass bottle compared to a natural environmental sample. Closed, static incubations may have altered mixing, grazing, and redox microgradients relative to the Mariager Fjord water column. For instance, cross-feeding on secondarily-produced ^13^C-substrates is also a consideration of any SIP experiment [47]. However, in seawater the concentration of dissolved inorganic carbon (DIC) is roughly 2 mM. This dilutes labeled carbon remineralized from the added substrates (in nanomolar concentrations) by several orders of magnitude [43]. Even though this huge dilution of the labeled inorganic carbon in the natural DIC pool reduces the likelihood of autotrophic cross-feeding, we do not know at what extent cross-feeding had occurred from ^13^C-organic byproducts. However, the relatively short incubation time was chosen to minimize the potential for recycling of organic ^13^C-labeled metabolites (e.g. due to cell death), which can increase with longer incubations [47]. Future work using controlled low-O_2_ incubators, biologically replicated designs, and real-time O_2_ monitoring will further improve our understanding of taurine and other organosulfur compounds in low-oxygen coastal environments.

### Organosulfur utilization under varying oxygen conditions

Microbial assimilation of ^13^C-taurine was undetectable under oxic conditions, where microbial communities displayed a clear uptake for ^13^C-glucose and ^13^C-methionine (Fig. 2 and 3). Methionine is a key sulfur [7] and carbon source [15] in the ocean, with its concentration varying with depth, diel cycle and phytoplankton activity [7]. Here, we showed that ASVs affiliated with *Haloferula* (*Verrucomicrobiota*), *Polaribacter* (*Flavobacteriaceae*), NS5 marine group (*Flavobacteriaceae*), and *Oleiphilus* (*Gammaproteobacteria*) were actively assimilating methionine in oxic incubations (Fig. 3A). These taxa, especially *Polaribacter* and NS5 marine group, are commonly associated with algal blooms and degradation of phytoplankton-derived organic matter [76]. Thus, our findings highlight an active, but previously unrecognized role for these carbon-assimilating taxa in methionine utilization.

In the hypoxic incubations, however, both ^13^C-DOS substrates tested in our study, ^13^C-taurine and ^13^C-methionine, were assimilated (Fig. 3C). Strong positive significant linear relationship was observed for ASVs assimilating ^13^C-taurine and ^13^C-methionine (Fig. 3C). This observation suggests a linked metabolic response in the uptake of organosulfur compounds. This relationship was not observed in marine sediments [15], suggesting that the processes controlling organosulfur assimilation in the water column of low-oxygen coastal systems versus benthic ecosystems may differ. The groups assimilating both ^13^C-methionine and ^13^C-taurine in Mariager Fjord were dominated by the *Flavobacteriaceae*, especially ASVs affiliated with *Ulvibacter*, *Lutibacter*, *Tenacibaculum*, NS4 marine group, unassigned genus under *Flavobacteriaceae*, as well as NS11–12 marine group (*Sphingobacteriales*) and one member under the order *Kapabacteriales*. Our study indicates that these groups, many of which are known for their ability to degrade high-molecular-weight organics [79], may also play a key role in the turnover of biolabile DOS compounds in seasonally stratified fjords.

Organosulfur compounds constitute a key source of carbon, sulfur, nitrogen and energy for marine microbial communities and may act as exchange currencies between phytoplankton and heterotrophic microbes [4, 5]. Although the cycling of biolabile DOS (e.g. DMS, DMSP) has been relatively well-studied in the oceanic photic zone [3–7], a detailed investigation on taxon-specific active microbial populations utilizing key organosulfur compounds such as taurine and methionine in low-oxygen environments was lacking. Mariager Fjord is a seasonally stratified coastal system with shallow depths, low salinity, and distinct microbial communities compared to open ocean OMZs. Our study quantifies the assimilation of ^13^C-taurine, ^13^C-methionine and ^13^C-glucose across an oxygen gradient in Mariager Fjord and shows that hypoxia increases the microbial assimilation of taurine, which is opposite to what has been observed in the open ocean [14]. This uptake was mainly performed by taxa within the *Bacteroidota*. In the hypoxic incubations, assimilation of ^13^C-taurine and ^13^C-methionine were strongly associated, indicating that certain microbes may be specialized in utilizing DOS as a carbon source under low-oxygen conditions. Altogether, our findings suggest a different pattern of organosulfur cycling in low-oxygen systems compared to open ocean settings, highlighting the distinct ecological dynamics of coastal hypoxic environments.

## Supplementary Material

Coskun_ISMEj_SI_submitted_wrag057

DataS1_qSIP_stats_v2_wrag057

DataS2_Density_Fractions_and_qPCR_values_wrag057

DataS3_Information_on_sequencing_data_wrag057

## Data Availability

All data needed to evaluate the paper are present in the main text or the supplementary materials. The intermediate files to replicate the study findings can be found in the Figshare repository under the following link: doi:10.6084/m9.figshare.29336066.v1. 16S rRNA sequences can be obtained from SAM using the accession numbers between SAMN49027424-SAMN49027683. The 16S rRNA amplicon sequences were entered in the NCBI Sequence Read Archive under BioProject ID PRJNA1271505.
