# Supplementary material for: Hypoxia increases microbial carbon assimilation of taurine in a seasonally anoxic fjord
Source: ISME J. 2026 Mar 17;20(1):wrag057. doi: 10.1093/ismejo/wrag057 (PMC13155104; doi:10.1093/ismejo/wrag057)
Supplement: Coskun_ISMEj_SI_submitted_wrag057 [file coskun_ismej_si_submitted_wrag057.docx]

**Supplementary Material**

**Hypoxia increases microbial carbon assimilation of taurine in a seasonally anoxic fjord**

Ömer K. Coskun^1*^, William D. Orsi^1,2^, Ian P. G. Marshall^3^, Katharina A. Muschler^1^, Nico Mitschke^4^, Timothy G. Ferdelman^5^, Gonzalo V. Gomez-Saez^1,2*^

**Affiliations:**

^1^ Department of Earth and Environmental Sciences, Ludwig-Maximilians-Universität München, Richard-Wagner-Straße 10, 80333 Munich, Germany

^2^ GeoBio-Center^LMU^, Ludwig-Maximilians-Universität München, Richard-Wagner-Straße 10, 80333 Munich, Germany

^3^ Section for Microbiology, Department of Biology, Aarhus University, Ny Munkegade 114, DK-8000 Aarhus C, Denmark

^4^ Institute for Chemistry and Biology of the Marine Environment (ICBM), School for Mathematics and Science, Carl von Ossietzky Universität Oldenburg, Ammerländer Heerstraße 114–118, 26129 Oldenburg, Germany

^5^ Department of Biogeochemistry, Max Planck Institute for Marine Microbiology, Celsius Straße 1, D-28359 Bremen, Germany

**^*^Corresponding authors:**

Dr. Ömer K. Coskun**,** Department of Earth and Environmental Sciences, Ludwig-Maximilians-Universität München, 80333 Munich, Germany. Email: [o.coskun@lrz.uni-muenchen.de](mailto:o.coskun@lrz.uni-muenchen.de)

Dr. Gonzalo V. Gomez-Saez, Department of Earth and Environmental Sciences, Ludwig-Maximilians-Universität München, 80333 Munich, Germany. Email: [g.gomez@lmu.de](mailto:g.gomez@lmu.de)

**Supplementary methods**

**Taurine measurements**

The pre-column derivatization was carried out in the auto-sampler at 7°C, where 1 µL of working solution to 4 µL of sample at 7°C and the derivatized sample was injected after 4 min reaction time. The autosampler working solution comprised 1 mL OPA reagent (0.27 g orthophthalaldehyde in 10 mL methanol), 3 mL borate buffer (0.3092 g boric acid in 50 mL MQ, pH 9.5) and 20 µL mercaptoethanol. Mobile phases were 20 mM sodium acetate (pH = 6.2) and acetonitrile-methanol solution (7.5% v/v) and thee flow rate was 0.35 mL min^-1^. The OPA-taurine derivative was separated on a 2.1x50 mm Acuity UPLC BEH-C18 1.7 µm column (Waters Corporation, Milford, MA, USA) at 30°C and eluted at 9.2 min. Excitation and emission wavelengths were 340 nm and 450 nm, respectively. Minimum detection limit based on taurine standards dissolved in low nutrient seawater (OSIL – Ocean Scientific International Ltd., Havant, U.K.) was 1.5 nM.

**Density gradient centrifugation and gradient fraction**

DNA samples were prepared for density gradient centrifugation according to previously published protocols for quantitative stable isotope probing (qSIP) with minor modifications [1, 2]. In brief, DNA was added to a mix solution of saturated cesium chloride (CsCl) and gradient buffer [0.1 M Tris-HCl (pH 8), 0.1 M KCl and 1 mM ethylenediaminetetraacetic acid (EDTA)] to reach an initial density of 1.69-1.70 g mL^-1^ in a 3.3 mL polyallomer OptiSeal tubes (Beckman Coulter, Brea, CA, USA). Density gradient centrifugations were performed in a TLN-100 Optima MAX-TL ultracentrifuge (Beckman Coulter, Brea, CA, USA) using a near-vertical rotor at 18 °C for 72 h at 165,000 x *g*. After centrifugation, the density gradients were fractioned into 20 equal fractions of 165 µL each by using a syringe pump and a fraction recovery system (Beckman Coulter, Brea, CA, USA). The density of the fractions was assessed by measuring refractive index of the DNA containing CsCl solution using an AR200 digital refractometer with temperature-corrected mode (nD-TC) (Reichert Analytical Instruments, Depew, NY, USA). Refractive index measurements for each CsCl aliquot were empirically standardized to density (g mL^-1^) using a conversion formula generated from calibration with CsCl/gradient buffer standards, as recommended in previous studies [3]. Retrieval of the DNA from the fractions were performed overnight at room temperature by adding two volumes polyethylene glycol (30% PEG 6000, 1.6 M NaCl) with 5 µL (5 mg mL^-1^) linear polyacrylamide (LPA). Following this, DNA was pelleted through centrifugation at 13,000 x *g* for 40 min, washed with 70% ethanol, and finally resuspended in 30-µL TE buffer (10 mM Tris-HCl and 1 mM EDTA, pH 8).

**Quantitative PCR analysis**

Quantitative PCR (qPCR) targeting the V4 hypervariable region of 16S ribosomal RNA (rRNA) was used to quantify the microbial abundances in all extracted DNA. A modified forward primer (515F-Y; 5′-GTG**Y**CAGCMGCCGCGGTAA) containing a single-base substitution was employed to enhance coverage across diverse bacterial and archaeal taxa [4]. For the reverse primer set, we used 806RB (5’-GGACTACNVGGGTWTCTAAT-3) to increase the detection of SAR11 targets [5]. All qPCRs were performed in a CFX Connect real-time PCR (Bio Rad, Hercules, CA, USA) as described previously [6]. Briefly, qPCRs were conducted in 20 µL volumes containing 10.4 µL SsoAdvanced SYBR Green PCR buffer (Bio-Rad, Hercules, CA, USA) using a two-step PCR protocol, including an initial step of enzyme activation at 95 °C for 3 min, followed by 40 cycles of denaturation at 95 °C for 15 s and annealing at 55 °C for 30 s. Standard curves for all assays were generated from 10-fold serial dilutions of purified PCR products amplified from representative samples using the same primer sets. Standards were gel-extracted, quantified with a Qubit fluorometer (Thermo Fisher Scientific, Waltham, MA, USA), and used to verify reaction efficiency. For each qPCR, a no-template control was added to assess contamination resulting from reagents itself.

**Further details on quantitative DNA stable-isotope-probing analysis and bioinformatics**

Quality filtering, read assembly, and contaminant removal from 16S rRNA gene amplicon sequences were carried out using established protocols described in [7] with minor modifications. In brief, amplicon sequence variants (ASVs) were generated using QIIME2 2024.5 distribution [8]. Raw sequence data were demultiplexed and quality filtered using q2-demux plugin followed by denoising with DADA2 [9] with default chimera removal settings (DataS3). To assign taxonomy to ASVs, we used “qiime feature-classifier classify-sklearn” naive Bayes taxonomy classifier [10] against SILVA 16S rRNA gene database (release 138.1; [11]). This database was downloaded from “https://data.qiime2.org/2024.2/common/silva-138-99-nb-classifier.qza”.

The excess ^13^C-atom fraction (EAF) values for each ASVs were calculated following qSIP previously described protocol [1] using the HTSSIP R package [12]. In brief, this approach combines sequencing data of each density fraction with qPCR to estimate isotopic enrichment at the taxon level. Initially, relative ASV abundances within each density fraction were converted to absolute abundances using total 16S rRNA gene copy numbers obtained from qPCR. Buoyant density (BD) shifts for each ASV (Z values) were then determined by calculating the differences between observed weighted average densities of ^13^C (W_lab_) and ^12^C (W_light_) treatments [1]. From these calculations, the ^13^C-EAF was computed for each ASV. EAF values of 0 indicate no detectable incorporation of ^13^C-labeled substrates, whereas EAF values of 1.0 represent complete (100%) labeling of the targeted V4 region of the 16S rRNA gene. Bootstrap confidence intervals (CIs) for isotope incorporation were generated using 1,000 bootstrap iterations in the HTSSIP R package. An ASV was considered a ^13^C incorporator if 90% lower confidence interval of its EAF value was greater than zero [1] (Data S1).

For principal coordinate analysis (PCoA), we downloaded multiple datasets obtained from NCBI sequence read archive (SRA) (Data S3). These included studies from the Saanich Inlet (Canada) [13], Roskilde Fjord (Denmark) [14], Kattegat Sea [15], Skagerrak Sea [15], Baltic Proper [15], Namibian Shelf [16], Golfo Dulce (Costa Rica) [17], and Indian Ocean [18]. In addition to these samples, we included in the PCoA unfractionated-SIP DNA data from 4 m and 138 m (4 m above bottom) depths from the Gotland Basin in the Baltic Sea (58°10´17.89´´N; 18°14´09.06´´E), representing oxic-to-anoxic conditions. Gotland Basin samples were obtained from the *RV* Skagerak expedition to the Baltic Sea from September 10 to October 3, 2023, led by University of Gothenburg (Sweden). On September 14, oxic and anoxic water samples were collected from two distinct Niskin bottles from a Sea-Bird SBE 32 rosette sampler. Aliquots (250 mL) were incubated with ^12^C- and ^13^C-labeled taurine, glucose, and methionine following the multisubstrate incubation protocol used for the Mariager Fjord dataset. Anoxic incubations were maintained at 4 °C, whereas oxic incubations were performed on board in the hangar at approximately 17 °C for 48 h. At the end of the incubation period, all samples were immediately frozen, stored at −20°C and shipped to LMU München for DNA extraction and sequencing. Accordingly, incubations were filtered in the lab using a 0.22 µm filters after thawing and DNA was concentrated using Amicon filters with 10 KDa pore size (Millipore, Merck, Darmstadt, Germany). Then, DNA was extracted following same protocol as Mariager Fjord samples. In the PCoA, additional lab contaminant sequences were obtained from previously published study [19]. Prior to QIIME2 analysis, these raw datasets were grouped based on which hypervariable region of 16S rRNA gene they represent. For example, studies which sequenced V4 hypervariable region of 16S rRNA gene were clustered into one group, namely Mariager Fjord (this study), Gotland Basin (this study), Namibian Shelf, Golfo Dulce, and lab contaminants. Then, each group was processed independently through the same qiime2 workflow (as explained previously) with their corresponding pre-trained classifier. In this way, we maintained consistency in quality filtering, denoising, and taxonomy assignments. Since ASVs produced from different hypervariable region of 16S rRNA gene could be directly compared to each other, each ASV table was then collapsed at genus level for cross-sample comparison. Samples were discarded if they have less than 1000 total reads. All datasets were combined into an abundance matrix and Bray-Curtis dissimilarity matrices were calculated using vegan packages (<https://github.com/vegandevs/vegan>) in R. Samples were annotated by a prepared metadata according to study origin and O_2_ conditions. To test for significant differences in community structure, permutational multivariate analyses of variance (PERMANOVA) were performed with adonis2(), using study site and O_2_ conditions as factors (999 permutations). The *R^2^* values reported from PERMANOVA represent the proportion of variation in community composition explained by the tested factor. Shannon diversity index as alpha diversity metric, was calculated using vegan’s estimateR() function and used to compare within- and between-study diversity. DataS3 was dedicated to present denoising stats resulting from DADA2, genus level matrix for the generation of PCoA, and results from alpha diversity metrics including Shannon index.

**Estimation of O_2_ diffusion from the incubation headspace**

To estimate the potential influence of O_2_ in the headspace on our incubations, we modeled O_2_ diffusion into incubated 1 L bottle during the SIP experiments using ChatGPT-assisted estimations (OpenAI). In brief, we have provided to the chatbot to model O_2_ diffusion into a non-agitated 1-L incubation bottles using Fick’s second law by providing the depth of the water (16.1 cm), initial O_2_ condition at the hypoxic layer (14 µM), O_2_ diffusion coefficient (*D*) obtained for 5 ^o^C [20] and microbial respiration rates from the literature [21].

Because the bottles were incubated without agitation, gas transfer from 160 mL atmospheric headspace into the water column occurs exclusively by molecular diffusion. Vertical O_2_ profiles were thus estimated using Fick’s second law with a zero-order microbial respiration term:

$$\frac{\partial C}{\partial t}=D\frac{\partial^{2}C}{\partial z^{2}}-R,$$

Where *C(z, t)* is the dissolved O_2_ concentration (µM) at depth *z* (m) and time *t* (s), *D* is the molecular diffusion coefficient of O_2_ in 5 ^o^C, *R* is the volumetric aerobic respiration rate (µM s^-1^). We used *D* = 1.36 x 10^-9^ m^2^ s^-1^ at 5 ^o^C seawater [20]. Initial O_2_ concentration is taken as the measured amount at the time of sampling (14 µM). We assumed that air-water interface has near saturation O_2_ concentration (352 µM). Microbial O_2_ respiration was taken from previously published rates in a nearby Danish fjord (30 and 400 nmol O_2_ L^-1^ h^-1^; [21]). The water depth that holds 1 L in our incubation settings was measured as 16.1 cm. The O_2_ diffusion was then simulated using one dimensional vertical grid (121 layers), solved by ChatGPT 5.1 with an explicit finite-difference scheme and a 300 s timesteps over a 48 h incubation. The results were taken ChatGPT and plotted in R studio using a chat-GPT assisted R script (see figshare repository). Dissolved O_2_ concentration at 48 h was used to obtain O_2_ distribution in vertical profile of the bottle (Fig. S3). At the water layer where O_2_ penetration is limited, we also assessed when exactly bottles experience anoxia, especially in the conditions where microbial respiration is high (Fig. S3B). These results and interpretations were used to revise manuscript’s text.

**Table S1: Summary of sequence reads across replicate sets within 12C- and ^13^C-labeled incubations and number of ASVs through quality-control steps.** A detailed explanation on data analyses is provided in the material and methods section of the main and the supplementary information.

**Figure S1: Density gradient shifts in DNA buoyant density between incubations with added ^12^C- and ^13^C-labeled substrates.** Quantification of 16S rRNA gene copies in CsCl density gradient fractions after 48 h of incubation with ^13^C-DOS and ^13^C-glucose. For each incubation, 16S rRNA gene abundances were normalized to the maximal DNA peak within replicate set. Colored solid lines with circles represent incubations with ^13^C-labeled substrates, whereas black dashed lines with squares represent control incubations (^12^C). Shaded boxes indicate the fractions that were selected for 16S rRNA gene amplicon sequencing in each replicate, and beige-filled circles mark additional fractions that were sequenced but fall outside of the main selection window. The weighted average densities for ^12^C- and ^13^C-incubations within each incubational setup are shown with triangles over the plots.

**Figure S2: Comparison of microbial communities in Mariager Fjord with publicly available datasets obtained from environmentally similar settings.** (A) Principal component analysis based on Bray-Curtis dissimilarities of microbial community abundances from original pre-incubation filters, qSIP incubations, and their comparison to datasets from seasonally low-oxygen fjords (Roskilde fjord, Saanich Inlet) and coastal shelf settings (Kattegat, Skagerrak, Golfo Dulce, Namibian shelf), and permanently hypoxic Gotland Basin. Colors represent the oxygen conditions of the samples, and shapes correspond to samples originating from the same environmental or experimental setting. (B) Shannon diversity index of the microbial communities. A detailed list of samples and matrices/tables used to produce both plots are provided in Supplementary methods and Data S3.

**Figure S3: Plots showing the diffusion of O_2_ from the headspace into the incubation water (A) after and (B) over 48 h of incubation.** Respiration rates are taken from previously published study in Randers Fjord in Denmark [21].

**Data S1: Spreadsheet showing qSIP results of the incubations amended with ^12^C- and ^13^C-labeled taurine, methionine and glucose along the oxygen gradients.**

**Data S2: Spreadsheet showing the density and starting 16S rRNA gene copy number of the fractions from three technical replicate sets. Sequenced density fractions are highlighted in their corresponding color.**

**Data S3: Spreadsheets summarizing the stats generated after DADA2 software embedded in qiime2 and genus level relative abundances across datasets used for FigS2. Metadata for PCoA and Shannon diversity index analyses are also included.**

Please find datasets as attached excel files to the Supplementary Material.

Table S1: Summary of sequence reads across replicate sets within unlabeled and ^13^C-labeled incubations and number of ASVs through quality-control steps. A detailed explanation on data analyses is provided in material and methods section in main and supplementary text.

| **Experiments** | **Total Sequence Reads** | **Percent of Contaminants** | **Total Contaminant Reads and < 10 reads** | **Clean Reads for Downstream Analysis** | **Number of ASVs (> 1 reads)** |
| --- | --- | --- | --- | --- | --- |
| Unlabeled incubation at oxic conditions - replicate 1 | 93731 | 2,96 | 2776 | 90955 | 144 |
| Unlabeled incubation at oxic conditions - replicate 2 | 104149 | 1,55 | 1614 | 102535 | 144 |
| Unlabeled incubation at oxic conditions - replicate 3 | 174378 | 1,79 | 3121 | 171257 | 239 |
| ^13^C-Glucose incubation at oxic conditions - replicate 1 | 205389 | 4,10 | 8417 | 196972 | 229 |
| ^13^C-Glucose incubation at oxic conditions - replicate 2 | 70172 | 2,14 | 1502 | 68670 | 137 |
| ^13^C-Glucose incubation at oxic conditions - replicate 3 | 70212 | 1,79 | 1259 | 68953 | 121 |
| ^13^C-Taurine incubation at oxic conditions - replicate 1 | 77533 | 1,26 | 979 | 76554 | 136 |
| ^13^C-Taurine incubation at oxic conditions - replicate 2 | 50401 | 1,12 | 567 | 49834 | 104 |
| ^13^C-Taurine incubation at oxic conditions - replicate 3 | 50765 | 4,42 | 2242 | 48523 | 214 |
| ^13^C-Methionine incubation at oxic conditions - replicate 1 | 118552 | 1,62 | 1921 | 116631 | 180 |
| ^13^C-Methionine incubation at oxic conditions - replicate 2 | 87554 | 3,13 | 2740 | 84814 | 161 |
| ^13^C-Methionine incubation at oxic conditions - replicate 3 | 74236 | 2,83 | 2098 | 72138 | 100 |
| Unlabeled incubation at hypoxic conditions - replicate 1 | 99657 | 4,04 | 4027 | 95630 | 190 |
| Unlabeled incubation at hypoxic conditions - replicate 2 | 80557 | 1,77 | 1425 | 79132 | 139 |
| Unlabeled incubation at hypoxic conditions - replicate 3 | 108579 | 1,08 | 1177 | 107402 | 118 |
| ^13^C-Glucose incubation at hypoxic conditions - replicate 1 | 163386 | 1,59 | 2592 | 160794 | 171 |
| ^13^C-Glucose incubation at hypoxic conditions - replicate 2 | 120642 | 4,90 | 5907 | 114735 | 285 |
| ^13^C-Glucose incubation at hypoxic conditions - replicate 3 | 224080 | 4,83 | 10812 | 213268 | 759 |
| ^13^C-Taurine incubation at hypoxic conditions - replicate 1 | 201777 | 8,24 | 16635 | 185142 | 574 |
| ^13^C-Taurine incubation at hypoxic conditions - replicate 2 | 197926 | 5,25 | 10392 | 187534 | 468 |
| ^13^C-Taurine incubation at hypoxic conditions - replicate 3 | 310942 | 3,33 | 10359 | 300583 | 560 |
| ^13^C-Methionine incubation at hypoxic conditions - replicate 1 | 383257 | 5,84 | 22373 | 360884 | 667 |
| ^13^C-Methionine incubation at hypoxic conditions - replicate 2 | 180205 | 4,14 | 7452 | 172753 | 371 |
| ^13^C-Methionine incubation at hypoxic conditions - replicate 3 | 268065 | 1,38 | 3711 | 264354 | 468 |
|  |  |  |  |  |  |
| **Quality-control step** | **Total number** |  |  |  |  |
| # ASVs (raw) | 6717 |  |  |  |  |
| # ASVs after >10 filter | 2270 |  |  |  |  |
| # ASVs after >10 filter + contaminant removal | 2100 |  |  |  |  |
| ^12/13^C-Glucose incubation at oxic conditions + "> 10 reads" in each replicate | 43 |  |  |  |  |
| ^12/13^C-Taurine incubation at oxic conditions + "> 10 reads" in each replicate | 42 |  |  |  |  |
| ^12/13^C-Methionine incubation at oxic conditions + "> 10 reads" in each replicate | 44 |  |  |  |  |
| ^12/13^C-Glucose incubation at hypoxic conditions + "> 10 reads" in each replicate | 84 |  |  |  |  |
| ^12/13^C-Taurine incubation at hypoxic conditions + "> 10 reads" in each replicate | 87 |  |  |  |  |
| ^12/13^C-Methionine incubation at hypoxic conditions + "> 10 reads" in each replicate | 86 |  |  |  |  |

**Figure S1: Density gradient shifts in DNA buoyant density between incubations with added ^12^C- and ^13^C-labeled substrates.**

Quantification of 16S rRNA gene copies in CsCl density gradient fractions after 48 h of incubation with ^13^C-DOS and ^13^C-glucose. For each incubation, 16S rRNA gene abundances were normalized to the maximal DNA peak within replicate set. Colored solid lines with circles represent incubations with ^13^C-labeled substrates, whereas black dashed lines with squares represent control incubations (^12^C). Shaded boxes indicate the fractions that were selected for 16S rRNA gene amplicon sequencing in each replicate, and beige-filled circles mark additional fractions that were sequenced but fall outside of the main selection window. The weighted average densities for ^12^C- and ^13^C-incubations within each incubational setup are shown with triangles over the plots.

**Figure S2: Comparison of microbial communities in Mariager Fjord with publicly available datasets obtained from environmentally similar settings.** (A) Principal component analysis based on Bray-Curtis dissimilarities of microbial community abundances from original pre-incubation filters, qSIP incubations, and their comparison to datasets from seasonally low-oxygen fjords (Roskilde fjord, Saanich Inlet) and coastal shelf settings (Kattegat, Skagerrak, Golfo Dulce, Namibian shelf), and permanently hypoxic Gotland Basin. Colors represent the oxygen conditions of the samples, and shapes correspond to samples originating from the same environmental or experimental setting. (B) Shannon diversity index of the microbial communities. A detailed list of samples and matrices/tables used to produce both plots are provided in Supplementary methods and Data S3.

**Figure S3: Plots showing the diffusion of O_2_ from the headspace into the incubation water (A) after and (B) over 48 h of incubation.** Respiration rates are taken from previously published study in Randers Fjord in Denmark [21].

**References**

1. Hungate BA, Mau RL, Schwartz E et al. Quantitative microbial ecology through stable isotope probing. Applied Environmental Microbiology. 2015;81:7570-81 <https://doi.org/10.1128/AEM.02280-15>

2. Coskun ÖK, Gomez-Saez GV, Beren M et al. Quantifying genome-specific carbon fixation in a 750-meter deep subsurface hydrothermal microbial community. FEMS Microbiology Ecology. 2024;100 <https://doi.org/10.1093/femsec/fiae062>

3. Neufeld JD, Vohra J, Dumont MG et al. DNA stable-isotope probing. Nature Protocols 2007;2:860-66 <https://doi.org/10.1038/nprot.2007.109>

4. Parada AE, Needham DM, Fuhrman JA. Every base matters: Assessing small subunit rRNA primers for marine microbiomes with mock communities, time series and global field samples. Environmental Microbiology. 2016;18:1403-14 <https://doi.org/https://doi.org/10.1111/1462-2920.13023>

5. Apprill A, McNally S, Parsons R, Weber L. Minor revision to V4 region SSU rRNA 806r gene primer greatly increases detection of SAR11 bacterioplankton. Aquatic Microbial Ecology. 2015;75:129-37

6. Coskun ÖK, Pichler M, Vargas S et al. Linking uncultivated microbial populations and benthic carbon turnover by using quantitative stable isotope probing. Applied Environmental Microbiology. 2018;84 <https://doi.org/10.1128/AEM.01083-18>

7. Coskun ÖK, Vuillemin A, Schubotz F et al. Quantifying the effects of hydrogen on carbon assimilation in a seafloor microbial community associated with ultramafic rocks. The ISME Journal. 2022;16:257-71 <https://doi.org/10.1038/s41396-021-01066-x>

8. Bolyen E, Rideout JR, Dillon MR et al. Reproducible, interactive, scalable and extensible microbiome data science using qiime 2. Nature Biotechnology. 2019;37:852-57 <https://doi.org/10.1038/s41587-019-0209-9>

9. Callahan BJ, McMurdie PJ, Rosen MJ et al. Dada2: High-resolution sample inference from illumina amplicon data. Nature Methods. 2016;13:581-83 <https://doi.org/10.1038/nmeth.3869>

10. Bokulich NA, Kaehler BD, Rideout JR et al. Optimizing taxonomic classification of marker-gene amplicon sequences with qiime 2’s q2-feature-classifier plugin. Microbiome. 2018;6:90 <https://doi.org/10.1186/s40168-018-0470-z>

11. Quast C, Pruesse E, Yilmaz P et al. The silva ribosomal RNA gene database project: Improved data processing and web-based tools. Nucleic Acids Res. 2013;41:D590-6 <https://doi.org/10.1093/nar/gks1219>

12. Youngblut ND, Barnett SE, Buckley DH. Htssip: An R package for analysis of high throughput sequencing data from nucleic acid stable isotope probing (SIP) experiments. PLOS ONE. 2018;13:e0189616 <https://doi.org/10.1371/journal.pone.0189616>

13. Hawley AK, Torres-Beltrán M, Zaikova E et al. A compendium of multi-omic sequence information from the Saanich Inlet water column. Scientific Data. 2017;4:170160 <https://doi.org/10.1038/sdata.2017.160>

14. Broman E, Asmala E, Carstensen J et al. Distinct coastal microbiome populations associated with autochthonous- and allochthonous-like dissolved organic matter. Frontiers Microbiology. 2019;10:2579 <https://doi.org/10.3389/fmicb.2019.02579>

15. Latz MAC, Andersson A, Brugel S et al. A comprehensive dataset on spatiotemporal variation of microbial plankton communities in the Baltic Sea. Scientific Data. 2024;11:18 <https://doi.org/10.1038/s41597-023-02825-5>

16. Vuillemin A, Coskun Ömer K, Orsi William D. Microbial activities and selection from surface ocean to subseafloor on the Namibian continental shelf. Applied and Environmental Microbiology. 2022;88:e00216-22 <https://doi.org/10.1128/aem.00216-22>

17. Steinsdóttir HGR, Gómez-Ramírez E, Mhatre S et al. Anaerobic methane oxidation in a coastal oxygen minimum zone: Spatial and temporal dynamics. Environmental Microbiology. 2022;24:2361-79 <https://doi.org/10.1111/1462-2920.16003>

18. Liu J, Cui Z, Luan X et al. Expanding oxygen minimum zones in the Northern Indian Ocean predicted by hypoxia-related bacteria. Frontiers in Marine Science. 2024;11:1396306 <https://doi.org/10.3389/fmars.2024.1396306>

19. Pichler M, Coskun ÖK, Ortega-Arbulú A-S et al. A 16S rRNA gene sequencing and analysis protocol for the Illumina Miniseq platform. MicrobiologyOpen. 2018:e00611 <https://doi.org/10.1002/mbo3.611>

20. Broecker WS, Peng T-H. Gas exchange rates between air and sea. Tellus. 1974;26:21-35 <https://doi.org/10.1111/j.2153-3490.1974.tb01948.x>

21. Holtappels M, Tiano L, Kalvelage T et al. Aquatic respiration rate measurements at low oxygen concentrations. PLOS ONE. 2014;9:e89369 <https://doi.org/10.1371/journal.pone.0089369>
